# Building a type 1 diabetes registry in South Africa: Insights from a national multi-stakeholder workshop

**DOI:** 10.4102/phcfm.v18i2.5580

**Published:** 2026-07-31

**Authors:** Patrick Ngassa Piotie, Penelope Modipane, Michelle Carrihill, Michelle Kruger, Marina Lazaridis, Adriaan Kruger, Luke Shankland, Elsabe Klinck, Camille Castelyn, Joseph Kibachio Mwangi, Melanie Pienaar, Mazvita Muchengeti

**Affiliations:** 1 School of Health Systems and Public Health, Faculty of Health Sciences, University of Pretoria, Pretoria, South Africa; 2 Department of Public Health, Medical School, Nelson Mandela University, Gqeberha, South Africa; 3 School of Medicine, Faculty of Health Sciences, University of Pretoria, Pretoria, South Africa; 4 Department of Paediatric Endocrinology, Faculty of Health Sciences, University of Cape Town, Cape Town, South Africa; 5 Nuvoteq, Pretoria, South Africa; 6 Aviro Health, Cape Town, South Africa; 7 Klinck and Samuels, Hermanus, South Africa; 8 Centre for Ethics and Philosophy of Health Sciences, Faculty of Health Sciences, University of Pretoria, Pretoria, South Africa; 9 World Health Organization, Pretoria, South Africa; 10 School of Nursing, Faculty of Health Sciences, University of the Free State, Bloemfontein, South Africa; 11 National Cancer Registry, National Health Laboratory Service, Johannesburg, South Africa; 12 Division of Epidemiology and Biostatistics, School of Public Health, University of the Witwatersrand, Johannesburg, South Africa; 13 South African DSI-NRF Centre of Excellence in Epidemiological Modelling and Analysis (SACEMA), Stellenbosch University, Stellenbosch, South Africa

## Introduction

Disease registries are widely recognised as essential public health tools for understanding disease burden, monitoring trends, improving quality of care and informing policy and resource allocation.^1,2^ In chronic conditions requiring lifelong management, registries play an important role by enabling longitudinal follow-up, identifying gaps in service delivery and supporting research and innovation.^3^ In the field of diabetes, registries have been instrumental in shaping clinical guidelines, access to technologies and health system planning in several high-income settings.^4,5^ However, the development of registries for specific diabetes subtypes, particularly type 1 diabetes (T1D), remains uneven globally and is especially limited in low- and middle-income countries.^6,7^ Type 1 diabetes presents distinct public health and health system challenges. It typically has an early age of onset, requires uninterrupted access to insulin and monitoring supplies and carries a high risk of acute and chronic complications when care is suboptimal.^8^ When T1D data are aggregated into broader diabetes datasets, the specific needs of children, adolescents and adults living with T1D risk are obscured. Dedicated T1D registries therefore serve a critical role in ensuring visibility, equity and accountability.^9^ In South Africa, the absence of reliable national data on T1D has been a longstanding concern for clinicians, researchers and patient advocacy organisations. Existing estimates are fragmented, often derived from small facility-based studies or extrapolated from other countries, and do not adequately reflect South Africa’s diverse population or health system. This data gap constrains effective planning, limits the ability to monitor progress towards national diabetes targets and weakens advocacy efforts aimed at improving access to care. Against this backdrop, a national workshop titled *Building a Direct-to-Participant Type 1 Diabetes Registry for South Africa* was convened during the 2025 Diabetes Summit. The workshop brought together a wide range of stakeholders to explore the feasibility, scope, governance and implementation considerations for establishing a national T1D registry. This conference report summarises the key discussions, areas of consensus and proposed next steps that emerged from the workshop.

## The South African context of type 1 diabetes and data scarcity

Despite increasing recognition of the growing burden of diabetes in South Africa, data on T1D remain particularly sparse.^10^ National surveillance systems do not currently distinguish reliably between diabetes subtypes, and routine health information systems are not designed to capture longitudinal outcomes that are specific to T1D. As a result, policymakers and health authorities lack reliable information on incidence, prevalence, geographic distribution, age of onset, glycaemic control, complications and mortality among people living with T1D. Recent research initiatives are addressing this gap. Notably, the South Africa T1D Burden Study, a multi-source epidemiological study currently underway in Gauteng and Free State, aims to generate baseline evidence on the T1D burden, clinical outcomes and care indicators. Using a hybrid recruitment approach that combines direct-to-participant online surveys with facility-based data collection supported by trained fieldworkers, this study demonstrated both the feasibility of national-scale data collection and the willingness of people living with T1D to contribute their data. Preliminary findings highlighted geographic disparities, limited reach within public sector facilities and frequent episodes of diabetic ketoacidosis, underscoring the need for sustained longitudinal surveillance. These emerging insights provide an important context for the registry workshop, reinforcing the urgency of moving beyond episodic studies towards a more systematic and sustainable approach to T1D surveillance in South Africa.

## Overview of the workshop: Purpose and approach

The workshop, held as part of the 2025 Diabetes Summit, was convened by the Diabetes Alliance South Africa in partnership with the nuvoteQ Foundation and Aviro Health. The session was designed as an interactive, multi-stakeholder dialogue. Participants included clinicians, epidemiologists, public health researchers, laboratory and data system experts, legal and ethics specialists, representatives of patient advocacy organisations and individuals living with T1D. The diversity of the participants reflected a deliberate effort to ensure that technical, ethical, clinical and lived experience perspectives informed discussions from the outset. The primary objective of the workshop was to explore the feasibility of establishing a national T1D registry in South Africa. The specific aims included clarifying the potential purpose and scope of such a registry, examining governance and legal requirements, identifying appropriate data sources and technical approaches and reflecting on lessons learned from previous registry efforts and existing models, including the National Cancer Registry.

## Key themes emerging from the workshop discussions

### Purpose and scope of a national type 1 diabetes registry

A central theme of the workshop was to define the primary purpose of a South African T1D registry. Participants discussed whether the registry should focus narrowly on epidemiological surveillance or adopt a broader scope that also captures psychosocial and socioeconomic data to support advocacy, service improvement and patient-centred care. While views varied, there was broad agreement that clarity of purpose is essential for sustainability and credibility. Participants cautioned against overly ambitious designs at inception and supported a phased approach, starting with a clearly defined core dataset aligned with surveillance needs and expanding incrementally as governance structures, funding and capacity evolve.

### Governance, ethics and legal considerations

Governance and ethics emerged as foundational considerations for any registry. Participants emphasised that registries are not neutral data systems. Instead, they shape visibility, participation and resource allocation, and therefore have ethical and social implications beyond technical data collection. From this perspective, ethical governance was framed as integral to registry design. Compliance with South Africa’s *Protection of Personal Information Act* (POPIA) was identified as non-negotiable, particularly given that T1D registries involve sensitive health data and include children and adolescents. The discussions highlighted the importance of transparent governance structures, clear data custodianship, defined access rules and the meaningful involvement of people living with T1D in oversight processes. The workshop also explored informed consent in the context of longitudinal registries. Participants recognised the limitations of one-off consent models and discussed the value of dynamic and ongoing consent approaches, including the use of multilingual, digital and offline formats, supported by sustained community engagement and feedback mechanisms. Ensuring long-term data stewardship and benefit sharing was considered critical for building and maintaining trust.

### Data sources, definitions and technical design

Participants explored potential data sources for a national registry, including patient-reported data, facility-based clinical records, laboratory data and medical scheme datasets. Workshop participants emphasised the value of data triangulation to improve case ascertainment and reduce duplication. A recurring concern was the absence of a standardised national definition of T1D, which increases the risk of misclassification and undermines data quality. Therefore, establishing clear clinical and operational definitions is an urgent priority. The discussion also considered hybrid data collection models that combine patient-entered data with healthcare worker support, balancing inclusivity, accuracy and feasibility.

### Equity, inclusion and lived experience

Equity and inclusion were prominent themes in the workshop. Participants stressed that a national T1D registry must actively address the barriers faced by people living in rural areas, those with limited digital access and individuals reliant on public sector services. Mobile data collection strategies, fieldworker support and partnerships with community organisations were identified as important enablers. Participants emphasised the importance of incorporating lived experience to ensure relevance and foster trust. However, participants cautioned that the inclusion of psychosocial and socioeconomic variables should be carefully aligned with the registry’s purpose to avoid overburdening people with T1D.

### Anticipated challenges and risks

Several challenges were identified, including funding sustainability, patient recruitment and retention, health system capacity constraints and potential hesitancy from health authorities given competing priorities and a preference for integrated non-communicable disease approaches. Participants agreed that addressing these challenges would require strong partnerships, incremental implementation and sustained advocacy. Recruitment and long-term engagement were highlighted as particularly significant implementation risks. Participants noted that beyond reducing barriers to participation, individuals living with T1D and their healthcare providers would need to perceive clear and ongoing value in contributing data over time, especially in resource-constrained settings. The workshop therefore emphasised the importance of a human-centred design approach to registry development, informed by principles of motivation and behaviour change, and grounded in continuous engagement with users. Uptake was framed as an ongoing relationship that must be supported through feedback, responsiveness and demonstrable benefit.

### Linking current research to future registry development

Insights from the ongoing South Africa T1D Burden Study were featured prominently in the workshop discussions. The study has already established a data collection infrastructure, governance approvals and multisectoral partnerships, providing a practical foundation for registry development. Importantly, the study demonstrated the feasibility of hybrid recruitment approaches and revealed high levels of trust and engagement within the diabetes community. Participants noted that these elements, namely trust, infrastructure and tested methodologies, are often the most difficult to establish and represent a significant opportunity to transition from a time-limited study to a sustainable, national registry.

## Conclusion

The workshop on building a T1D registry in South Africa marked an important moment in the national efforts to address longstanding data gaps. Through open, multi-stakeholder dialogue, participants articulated a shared recognition that people living with T1D remain insufficiently visible within current health information systems and that this invisibility has tangible consequences for care, policy and outcomes. While significant challenges remain, the workshop demonstrated a growing convergence around the need for a phased, pragmatic and ethically grounded approach to developing a registry. Ethical governance, dynamic consent, transparency and patient participation were not viewed as obstacles but as enablers of trust and sustainability. Emerging research initiatives, strong community engagement and lessons learned from existing registry models provide a solid foundation for establishing a national T1D registry. Ultimately, a national T1D registry has the potential not only to improve surveillance and inform policy, but also to affirm the principle that every person living with T1D in South Africa is important and deserves to be seen.
